# Supplementary material for: Efficient gene disruption with CRISPR–Cas3 in human T cells
Source: NAR Cancer. 2026 Apr 21;8(2):zcag009. doi: 10.1093/narcan/zcag009 (PMC13137050; doi:10.1093/narcan/zcag009)
Supplement: zcag009_Supplemental_Files [file zcag009_supplemental_files.zip › 260225_Supplementary Figures.pdf]

Supplementary Figure legends

Figure S1. Schematic overview of the experimental procedure for genome editing in human primary T cells. PBMCs were activated with anti-CD3 antibody (OKT3), Retronectin, and IL-2 for three days, followed by electroporation with Cas3-Cascade mRNAs and crRNAs. Disruption efficiency, cell viability, and live cell number were analyzed on day 7.

Figure S2. Off-target analysis in human primary T cells introduced with the CRISPR-Cas9 mRNA. (A) Cells edited with Cas9_TRAC sgRNA. (B) Cells edited with Cas9_B2M sgRNA. (C, D) IGV images showing edited (top) and control (bottom) sequencing reads at off-target sites identified in *TRAC*-edited (C) or *B2M*-edited (D) human primary T cells. Detected off-target loci include the *ECT2L* gene locus on chromosome 6 and a locus on chromosome 14, highlighting the potential for unintended editing by CRISPR-Cas9.

Figure S3. Optimization of genome editing in PBMCs using CRISPR-Cas3 mRNA under modified culture conditions. (A, B) PBMCs were activated for 3 days in RPMI 1640 medium supplemented with 10% FBS in the presence of anti-CD3 antibody (OKT3), Retronectin, and IL-2. A total of 1.0 × 106 activated cells were electroporated with Cas3-Cascade mRNAs (1.0 µg each) together with either B2M or TRAC crRNA (1.6 µg) to induce single-gene disruption. Genome editing efficiency (B, left) and cell viability (B, right) were evaluated by flow cytometry on day 7. (C, D) PBMCs stimulated under the same conditions were electroporated with Cas3-Cascade mRNAs (1.0 µg each) together with both B2M and TRAC crRNAs (0.8 µg each) to achieve simultaneous knockout of

B2M and TRAC. Genome editing efficiency of each gene (D, left) and cell viability (D, right) were estimated by flow cytometry on day 7. Error bars represent mean ± SEM (n = 3).

Figure S4. (A) Schematic representation of CRISPR–Cas3 mRNAs (see Table S10). Gray regions indicate the bipartite nuclear localization signal (bpNLS), and black regions indicate the coding sequences of each Cas protein. (B) Structure and chemical modifications of the crRNA (see Table S1). Bold letters indicate 2′-O-methyl–modified nucleotides, and asterisks indicate phosphorothioate linkages.

Figure S1

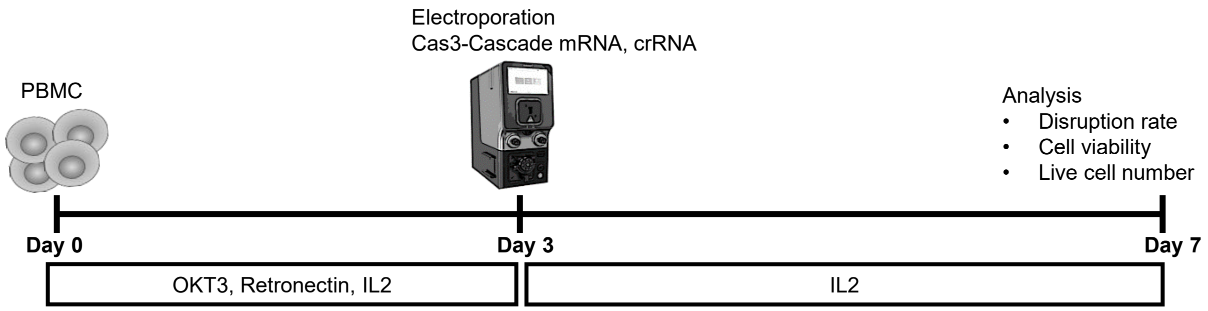
Electroporation
Cas3-Cascade mRNA, crRNA
PBMC
Analysis
• Disruption rate
• Cell viability
• Live cell number
Day 0
Day 3
Day 7
OKT3, Retronectin, IL2
IL2

Figure S2

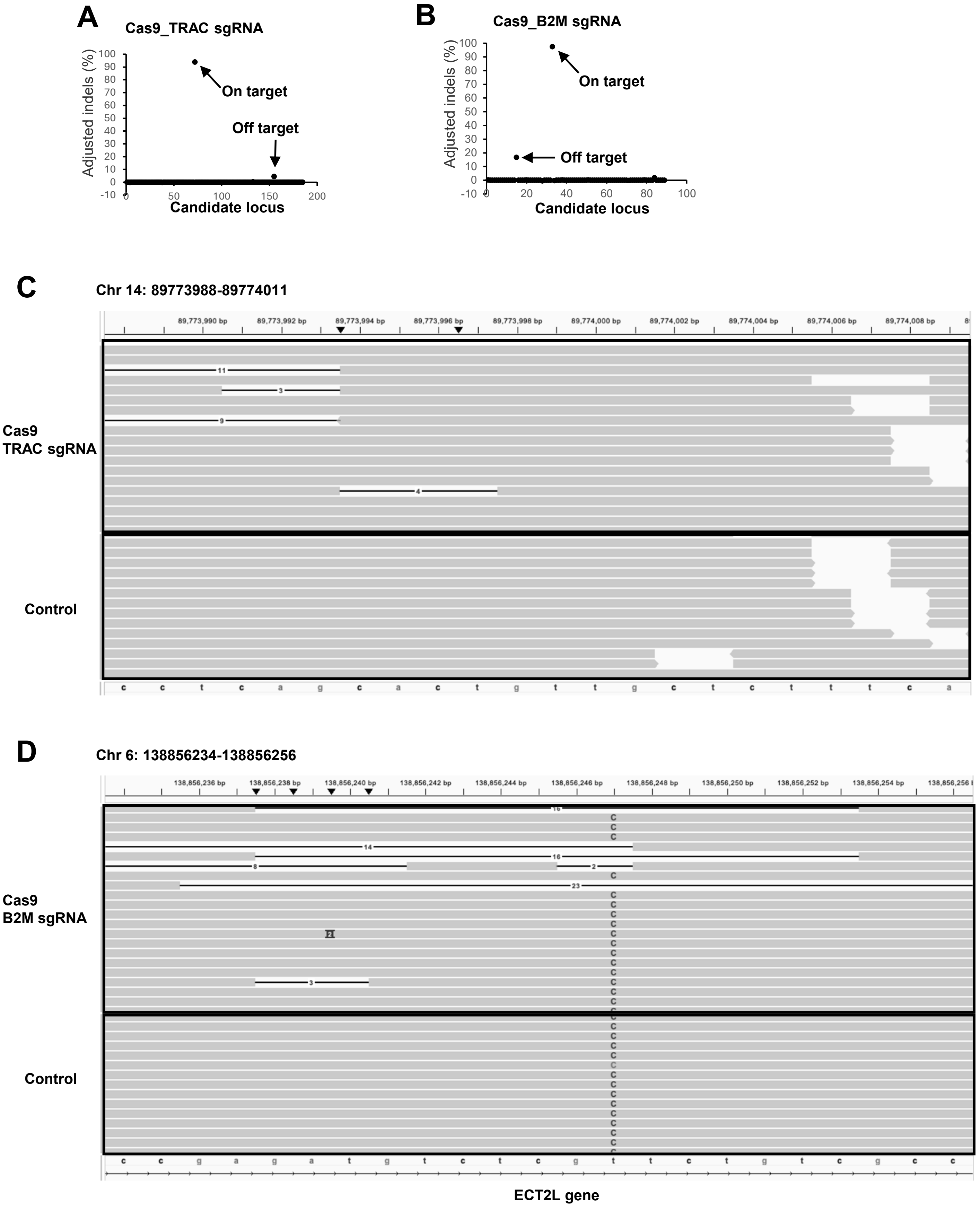
A
Cas9_TRAC sgRNA
Adjusted indels (%)
On target
Off target
Candidate locus
B
Cas9_B2M sgRNA
Adjusted indels (%)
On target
Off target
Candidate locus
C
Chr 14: 89773988-89774011
Cas9
TRAC sgRNA
Control
D
Chr 6: 138856234-138856256
Cas9
B2M sgRNA
Control
ECT2L gene

Figure S3

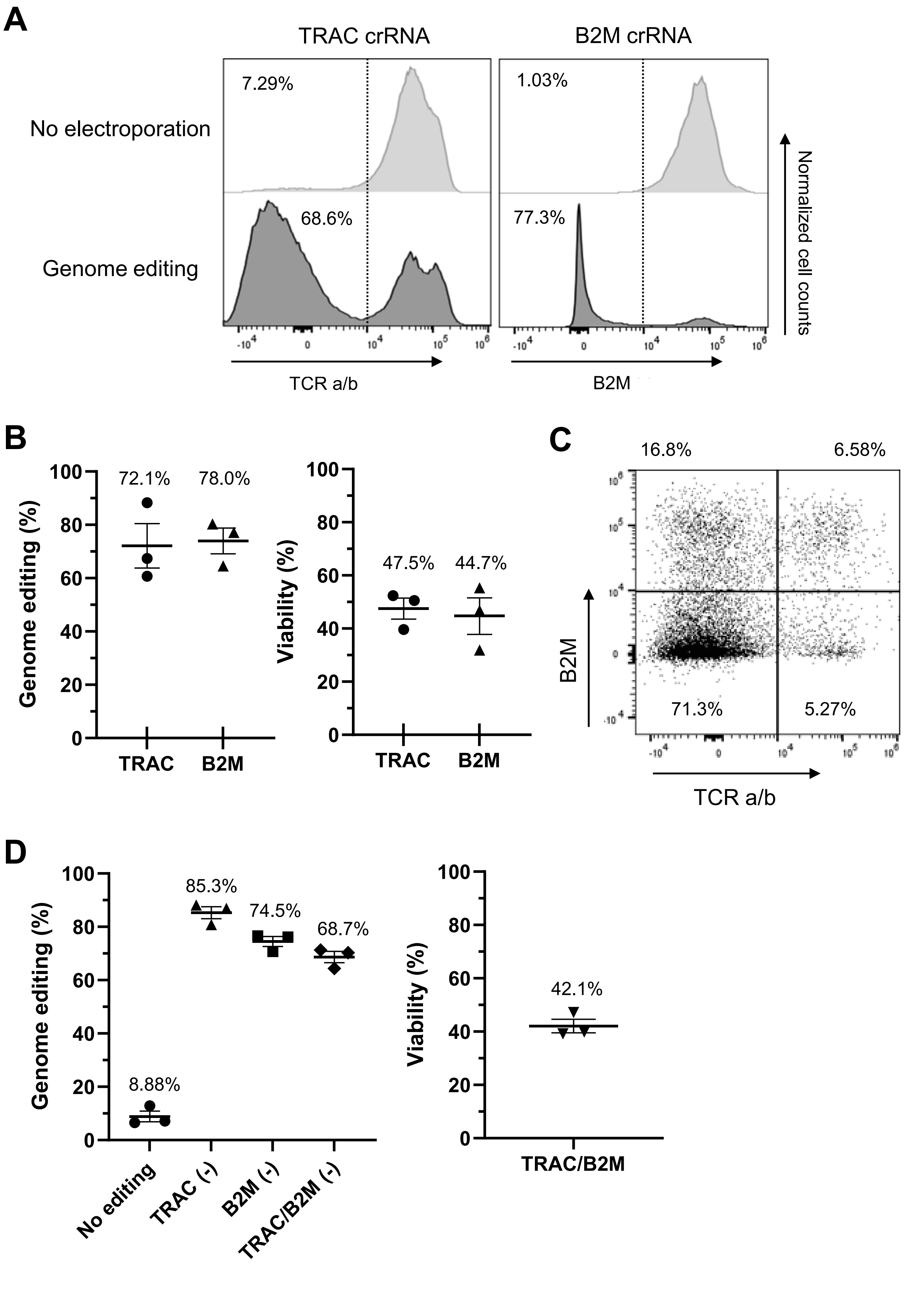

A
TRAC crRNA
B2M crRNA
No electroporation
Genome editing
7.29%
68.6%
1.03%
77.3%
Normalized cell counts
TCR a/b
B2M
B
Genome editing (%)
72.1%
78.0%
TRAC
B2M
Viability (%)
47.5%
44.7%
TRAC
B2M
C
16.8%
6.58%
71.3%
5.27%
B2M
TCR a/b
D
Genome editing (%)
8.88%
85.3%
74.5%
68.7%
No editing
TRAC (-)
B2M (-)
TRAC/B2M (-)
Viability (%)
42.1%
TRAC/B2M

Figure S4

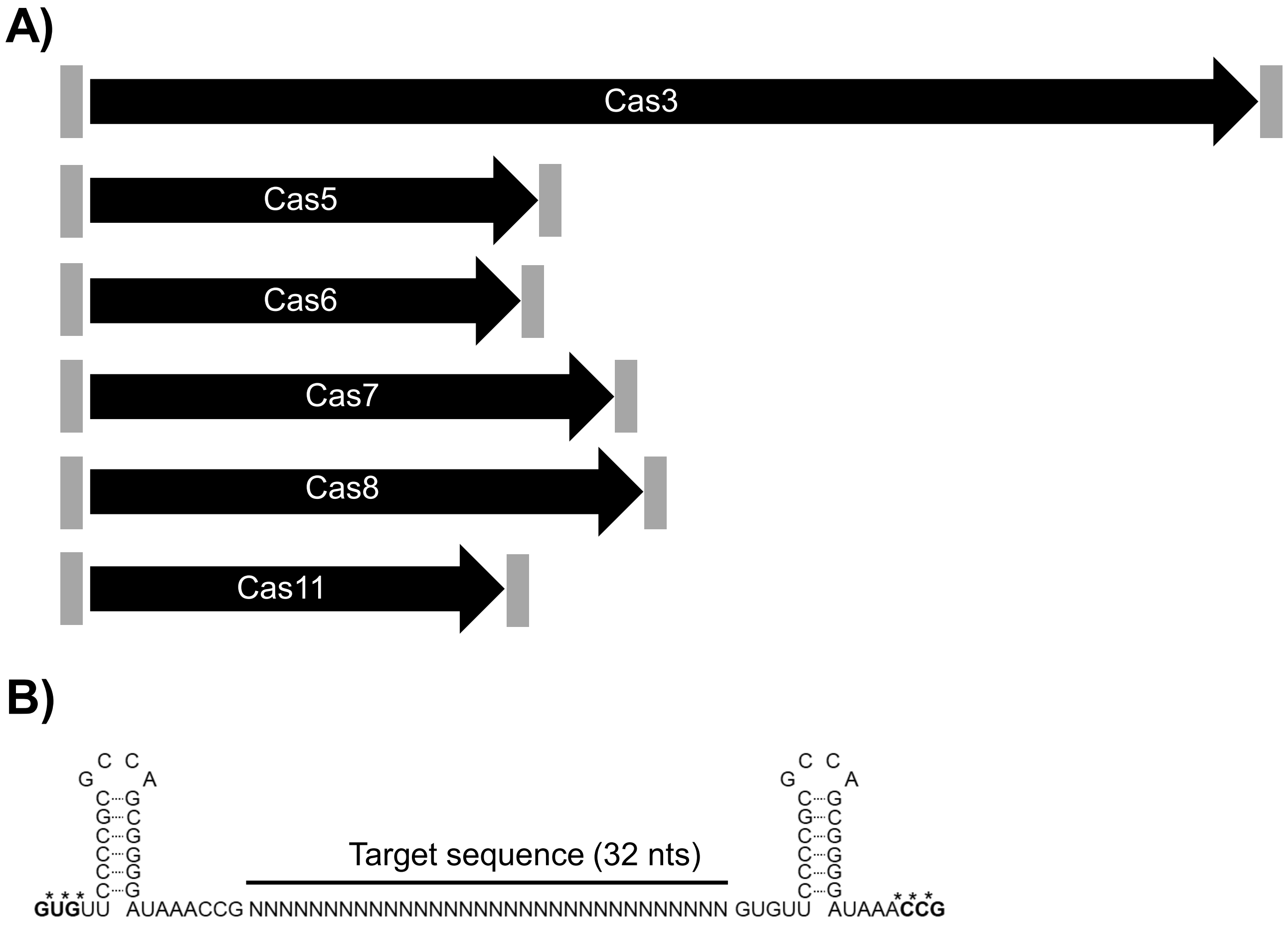
A)
Cas3
Cas5
Cas6
Cas7
Cas8
Cas11
B)
Target sequence (32 nts)
GUGUU AUAAACCG NNNNNNNNNNNNNNNNNNNNNNNNNNNNNNNN GUGUU AUAAACCG
